# Antibiotics Associated With Clostridium difficile Infection

**DOI:** 10.7759/cureus.39029

**Published:** 2023-05-15

**Authors:** Abdur Rafey, Shah Jahan, Umer Farooq, Furqana Akhtar, Memoona Irshad, Summiya Nizamuddin, Azra Parveen

**Affiliations:** 1 Department of Internal Medicine and Infectious Diseases, Shaukat Khanum Memorial Cancer Hospital and Research Center, Lahore, PAK; 2 Department of Internal Medicine, Shaukat Khanum Memorial Cancer Hospital and Research Center, Lahore, PAK; 3 Department of Infectious Diseases, Bahria International Hospital, Lahore, PAK; 4 Department of Infectious Diseases, Aga Khan University Hospital, Karachi, PAK; 5 Department of Microbiology, Shaukat Khanum Memorial Cancer Hospital and Research Center, Lahore, PAK

**Keywords:** levofloxacin, ceftriaxone, ciprofloxacin, vancomycin, meropenem, piperacillin/tazobactam, clostridium difficile

## Abstract

Introduction

*Clostridium difficile* (*C. difficile*) is one of the major causes of diarrhea transmitted by the fecal-oral route. *C. difficile* type BI/NAP1/027 is responsible for the most severe *C. difficile* infection (CDI). It is a major cause of antibiotic-associated diarrhea followed by *Clostridium perfringens, Staphylococcus aureus,*and* Klebsiella oxytoca. *Historically, clindamycin, cephalosporins, penicillins, and fluoroquinolones were related to CDI. We conducted this study to evaluate the antibiotics associated with CDI in recent times.

Methods

We conducted a retrospective, single-center study over a period of eight years. A total of 58 patients were enrolled in the study. Patients with diarrhea and positive *C. difficile* toxin in stool were evaluated for antibiotics given, age, presence of malignancy, previous hospital stay for more than three days in the last three months, and any comorbidities.

Results

Among patients who developed CDI, prior antibiotics for at least four days duration were given in 93% (54/58) of patients. The most common antibiotics associated with *C. difficile* infection were piperacillin/tazobactam in 77.60% (45/58), meropenem in 27.60% (16/58), vancomycin in 20.70% (12/58), ciprofloxacin in 17.20% (10/58), ceftriaxone in 16% (9/58), and levofloxacin in 14% (8/58) of patients, respectively. Seven percent (7%) of patients with CDI did not receive any prior antibiotics. Solid organ malignancy was present in 67.20% and hematological malignancy in 27.60% of CDI patients. Ninety-eight percent (98%, 57/58) of patients treated with proton pump inhibitors, 93% of patients with a previous hospital stay for more than three days, 24% of patients with neutropenia, 20.1% of patients aged more than 65 years, 14% of patients with diabetes mellitus, and 12% of patients with chronic kidney disease also developed *C. difficile* infection.

Conclusion

The antibiotics associated with *C. difficile* infection are piperacillin/tazobactam, meropenem, vancomycin, ciprofloxacin, ceftriaxone, and levofloxacin. Other risk factors for CDI are proton pump inhibitor use, prior hospital admission, solid organ malignancy, neutropenia, diabetes mellitus (DM), and chronic kidney disease (CKD).

## Introduction

*Clostridioides difficile* was previously known as *Clostridium difficile (C. difficile)*. It is one of the major causes of diarrhea in the Western world [1] and is a Gram-positive, anaerobic, toxin-producing bacteria whose spores are transmitted by the fecal-oral route. The main protective barrier against *C. difficile* infection (CDI) is the normal indigenous intestinal microflora [2-4]. Bile acids play an important role in spore germination [4]. Virulence factors are enzymes including collagenase, hyaluronidase, chondroitin-sulfatase, and two types of toxins, A and B, causing intestinal damage [3,5]. *C. difficile* type BI/NAP1/027 is resistant to fluoroquinolones and causes the most severe CDI [6,7].

The role of *C. difficile* in antibiotic (clindamycin, cephalosporins, ampicillin, amoxicillin, tetracycline, and ciprofloxacin) -associated pseudomembranous colitis dates back to the 1970s [2,8-11]. The most common cephalosporins associated with CDI were cefotaxime and ceftriaxone [9,10]. Reduced prescription of these injectable cephalosporins [12,13] and levofloxacin [14] is a cost-effective method of reducing the incidence of *C. difficile-associated* diarrhea. Increased risk of CDI is associated with cumulative dose, number of antibiotics, and days of antibiotic exposure [15,16].

Bacteria other than *C difficile* that cause antibiotic-associated diarrhea are *Clostridium perfringens*, *Staphylococcus aureus,* and *Klebsiella oxytoca* [17].

Limited data are available regarding CDI in Asia. Recently rates of CDI in Asia are becoming equal to Europe and North America [18].

We conducted this study to evaluate recent antibiotics associated with a *C. difficile* infection in Pakistan.

## Materials and methods

We conducted a retrospective, single-center study at Shaukat Khanum Memorial Cancer Hospital and Research Center (SKMCH&RC), which is a tertiary care cancer center in Lahore, Pakistan. We included patients over a period of eight years from January 1, 2015, to September 30, 2022, and reviewed their medical records. This study was approved by the Institutional Review Board at SKMCH&RC with a waiver of informed consent. We included all patients (all ages and both inpatients and outpatients) with diarrhea and positive *C. difficile* toxin in stool, detected via nucleic acid amplification using a real-time polymerase chain reaction (PCR) assay (the GeneXpert technique). We evaluated patients regarding antibiotics given for at least four days [15] in the last three months and features of severe infection or not [19]. We also evaluated patients for other characteristics, including age, type of malignancy, presence of neutropenia, previous hospital stay for more than three days in the last three months, and any comorbidities. Frequencies and proportions were reported for categorical variables. Mean and standard deviation was reported for continuous variables.

## Results

A total of 58 patients were evaluated in the study, further supporting the rarity of *C. difficile *infection in the Asian subcontinent [20]. There were 79.3% (46/58) male and 20.7% (12/58) female patients. There were 62% (36/58) adults and 38% (22/58) children. The mean age was 30 years with a standard deviation of + 56. Solid organ malignancy was present in 67.20% (39/58) of patients, hematological malignancy was found in 27.60% (16/58), and 5.20% (3/58) were non-cancer patients. Thirty-eight percent (38%, 22/58) of patients had severe CDI. Results are summarized in Table 1.

**Table 1:** Demographic characteristics CA - cancer, SCC - squamous cell carcinoma, ALL - Pre B ALL - pre-B acute lymphoblastic leukemia, DLBCL - diffuse large B cell lymphoma, MM - multiple myeloma, HL - Hodgkin lymphoma

	n(%)
Male	46/58(79.30%)
Female	12/58(20.70%)
Adults	36/58(62%)
Children	22/58(38%)
Solid organ cancer	39/58(67.20%)
CA breast	8/39(21%)
CA colon	5/39(12.80%)
CA prostate	4/39(10%)
CA ovary	4/39(10%)
SSC oral cavity	4/39(10%)
CA bladder	3/39(8%)
CA stomach	2/39(5%)
CA cervix	2/39(5%)
Ewing sarcoma	2/39(5%)
Esophageal CA	1/39(2.60%)
CA pancreas	1/39(2.60%)
Wilm`s tumor	1/39(2.60%)
Seminoma	1/39(2.60%)
CA larynx	1/39(2.60%)
Hematological cancer	16/58(27.60%)
Burkitt lymphoma	5/16(31.20%)
Pre B ALL	5/16(31.20%)
DLBCL	2/16(12.50%)
MM	2/16(12.50%)
HL	1/16(6.20%)
B cell lymphoma	1/16(6.20%)
Non-cancer patients	3/58(5.20%)

Prior antibiotics for at least four days duration in the last three months were given in 93% (54/58) of patients. The most common antibiotics given in patients with *C. difficile* infection were piperacillin/tazobactam given in 77.60% (45/58), followed by meropenem given in 27.60% (16/58), vancomycin given in 20.70% (12/58), ciprofloxacin given in 17.20% (10/58), ceftriaxone given in 16% (9/58) and levofloxacin given in 14% (8/58) of patients respectively. These antibiotics were given in recommended standard doses and adjusted for renal or hepatic failure if present. Results are summarized in Table 2.

**Table 2:** Antibiotics associated with C. difficile infection

	n(%)
Prior antibiotic use	54/58(93%)
Piperacillin/tazobactum	45/58(77.60%)
Meropenem	16/58(27.60%)
Vancomycin	12/58(20.70%)
Ciprofloxacin	10/58(17.20%)
Ceftriaxone	9/58(16%)
Levofloxacin	8/58(14%)
Co-amoxiclav	7/58(12%)
Colistimathate sodium	6/58(10.30%)
Clindamycin	5/58(8.60%)
Ertapenem	5/58(8.60%)
Imipenem	3/58(5.20%)
Azithromycin	3/58(5.20%)
Teicoplanin	2/58(3.40%)
Fosfomycin	2/58(3.40%)
Cephalexin	2/58(3.40%)
Moxifloxacin	2/58(3.40%)
Linezolid	2/58(3.40%)
Amikacin	2/58(3.40%)
Nitrofurantoin	1/58(2%)
Cephazoline	1/58(2%)

Other than antibiotic exposure, 98% (57/58) of patients treated with proton pump inhibitors in standard dosing, 93% (54/58) of patients with a previous hospital stay for more than three days in the last three months, 24% (14/58) of patients with neutropenia, 20.1% (12/58) of patients aged more than 65, 14% (8/58) of patients with diabetes mellitus, and 12% (7/58) of patients with chronic kidney disease also developed *C. difficile* infection. Results are summarized in Table 3.

**Table 3:** Risk factors for CDI other than antibiotics PPI - proton pump inhibitors, DM - diabetes mellitus, CKD - chronic kidney disease, IHD - ischemic heart disease, HTN - hypertension, NGT - nasogastric tube, ICU - intensive care unit

	n(%)
PPI use	57/58(98%)
Prior admission	54/58(93%)
Neutropenia	14/58(24%)
Age > 65	12/58(20.1%)
Methotrexate	10/58(17%)
Surgery	10/58(17%)
DM	8/58(14%)
CKD	7/58(12%)
IHD	4/58(7%)
HTN	4/58(7%)
NGT	4/58(7%)
ICU admission	3/58(5%)

## Discussion

The results of our study showed that the most common antibiotics associated with *C. difficile* infection were piperacillin/tazobactam, meropenem, vancomycin, ciprofloxacin, ceftriaxone, and levofloxacin. Seven percent (7%, 4/58) of patients with CDI did not receive any prior antibiotics. Males were affected more than females and adults were affected more than children. Most CDI occurred in patients with solid organ tumors and neutropenia (14%, 14/58). The use of proton pump inhibitors and prior hospital admission was the most common risk factors for CDI, other than antibiotics exposure.

Our study supports the results of previous studies that prior admission for more than three days [21], use of antibiotics [2,8-11], including piperacillin-tazobactam [22], carbapenems [16,21], ciprofloxacin [9], penicillins [2] and proton pump inhibitors [22,23], is associated with CDI.

A review of the literature also showed that solid organ malignancy and methotrexate use were also risk factors for CDI [16,24] without prior antibiotic treatment. However, in our study, methotrexate was given to 17% (10/58) of patients with CDI. Old age is also a risk factor [21,25] but in our results, only 20.1% (12/58) of elderly patients developed CDI. Diabetes mellitus [22,26] and chronic kidney disease (CKD) [16,27] are also risk factors for CDI but only 14% (8/58) had diabetes and 12% (7/58) had CKD in our results. Intra-abdominal surgery, nasogastric tube use, and intensive care unit (ICU) stay [16,27] are also risk factors for CDI that were present in 17% (10/58), 7% (4/58), and 5% (3/58), respectively, in our study population.

The major strengths of our study are its duration and the highlighting of a complex issue often neglected in complicated hospitalized patients in Pakistan.

As our study population consisted of complicated cancer patients on treatment as well as other complications, the risk of CDI was increased not only because of antibiotics use but also because of malignancy, age, comorbidities, and surgery, thus confounding the culprit antibiotics. The major weakness of our study is its retrospective single-center design with a relatively small sample size. We recommend multicenter retrospective or prospective cohort studies for further research purposes.

## Conclusions

The most common antibiotics associated with *C. difficile* infection are piperacillin/tazobactam, meropenem, vancomycin, ciprofloxacin, ceftriaxone, and levofloxacin. Few patients with CDI did not receive any prior antibiotics but had other risk factors of proton pump inhibitor use, prior hospital admission, solid organ malignancy, neutropenia, DM, and CKD. If patients with these risk factors develop diarrhea, they should be evaluated for CDI.
